# The Composite Effect of Transgenic Plant Volatiles for Acquired Immunity to Herbivory Caused by Inter-Plant Communications

**DOI:** 10.1371/journal.pone.0024594

**Published:** 2011-10-12

**Authors:** Atsushi Muroi, Abdelaziz Ramadan, Masahiro Nishihara, Masaki Yamamoto, Rika Ozawa, Junji Takabayashi, Gen-ichiro Arimura

**Affiliations:** 1 Global COE Program: Evolution and Biodiversity, Graduate School of Science, Kyoto University, Kyoto, Japan; 2 Center for Ecological Research, Kyoto University, Otsu, Japan; 3 Botany Department, Faculty of Science, Ain Shams University, Cairo, Egypt; 4 Iwate Biotechnology Research Center, Kitakami, Iwate, Japan; AgroParisTech, France

## Abstract

A blend of volatile organic compounds (VOCs) emitted from plants induced by herbivory enables the priming of defensive responses in neighboring plants. These effects may provide insights useful for pest control achieved with transgenic-plant-emitted volatiles. We therefore investigated, under both laboratory and greenhouse conditions, the priming of defense responses in plants (lima bean and corn) by exposing them to transgenic-plant-volatiles (VOCos) including (*E*)-β-ocimene, emitted from transgenic tobacco plants (NtOS2) that were constitutively overexpressing (*E*)-β-ocimene synthase. When lima bean plants that had previously been placed downwind of NtOS2 in an open-flow tunnel were infested by spider mites, they were more defensive to spider mites and more attractive to predatory mites, in comparison to the infested plants that had been placed downwind of wild-type tobacco plants. This was similarly observed when the NtOS2-downwind maize plants were infested with *Mythimna separata* larvae, resulting in reduced larval growth and greater attraction of parasitic wasps (*Cotesia kariyai*). In a greenhouse experiment, we also found that lima bean plants (VOCos-receiver plants) placed near NtOS2 were more attractive when damaged by spider mites, in comparison to the infested plants that had been placed near the wild-type plants. More intriguingly, VOCs emitted from infested VOCos-receiver plants affected their conspecific neighboring plants to prime indirect defenses in response to herbivory. Altogether, these data suggest that transgenic-plant-emitted volatiles can enhance the ability to prime indirect defenses via both plant-plant and plant-plant-plant communications.

## Introduction

In response to herbivory, plants start to defend themselves against herbivores by producing toxins, repellents, antinutritives, etc. (induced direct defense), and by emitting a specific blend of volatile organic compounds (VOCs) that attract the carnivorous natural enemies of herbivores (induced indirect defense) [1]. Along with gaseous phytohormones (e.g., ethylene) induced by herbivory, VOCs including a wide array of low molecular weight terpenes and green leaf volatiles function as airborne signals within and between plants [2]–[8]. Such signals allow receiver plants to tailor their defenses to their current and expected risks caused by herbivores. On occasion, receiver plants do not show immediate changes in their level of defenses, but respond stronger and faster than non-receiver plants when damaged by herbivores [7], [9]–[13]. This readying of a defense response, termed ‘priming’, is demonstrated by the fact that volatiles emitted from clipped sagebrush (*Artimisia tridentata*) affected neighboring *Nicotiana attenuata* plants by accelerating production of trypsin proteinase inhibitors only after *Manduca sexta* larvae started to attack [12]. In hybrid poplar, the expression of genes involved in direct defense was not highly induced in the leaves exposed to one of the green leaf volatiles, (*Z*)-3-hexen-1-yl acetate (Hex-Ac), before herbivory, but was strongly induced once herbivores (gypsy moth larvae) began to feed [10]. Such priming effects were similarly observed in maize plants which had been exposed to VOCs emitted from maize plants infested with generalist herbivores [11]. *Spodoptera littoralis* did not activate genes that are responsive to wounding, jasmonic acid, or caterpillar regurgitant, but showed primed expression of these genes and reduced caterpillar feeding and development [11]. Exposure to the volatiles also enhanced the emission of volatiles in receiver plants that could attract carnivorous natural enemies, which could help the plants' indirect defense [11].

There are also a few field studies showing similar effects. Wild tobacco plants that were growing near experimentally clipped sagebrush plants showed increased ability to respond to herbivore attack and received less damage over the growing season [12], [14]. Similarly, wild lima bean shoots responded to the volatile cues released by conspecifics that were experimentally exposed to beetle feeding by increasing several direct and indirect defenses [7]. Tendrils induced by eavesdropping on airborne emissions of neighbors produced more leaves and inflorescences than uninduced controls. Further, the VOCs can prime extrafloral nectar secretion, a taxonomically widespread anti-herbivore defense [15].

One potential approach to understanding volatile communication involves using transgenic or mutant plants that are genetically modified in their potential to emit or receive VOC signals. In the current study, we used transgenic tobacco plants emitting (*E*)-β-ocimene [(3*E*)-3,7-dimethyl-l,3,6-octatriene] as emitters for plant-plant communication assays. The acyclic monoterpene hydrocarbon β-ocimene was recently found to induce increased tissue levels of methyl jasmonate and transcript levels of defense/stress-inducible genes in Arabidopsis [16]. Furthermore, transgenic Arabidopsis plants expressing a GUS-reporter gene under the control of the potato proteinase inhibitor II promoter (*pinII*) responded to several structurally different cyclic and acyclic monoterpenes (including β-ocimene) [16]. This is in line with the finding that six volatile terpenes increase the cytoplasmic free Ca^2+^ concentration in Arabidopsis leaf cells in a similar transient fashion [17]. Physicochemical processes, including interactions with odorant binding proteins and resulting in changes in transmembrane potentials, can underlie VOCs-mediated signaling processes [18]. However, whether the volatile responses occur in a specific fashion remains to be answered.

In our experiments, dicotyledon and monocotyledon crops (bean and maize, respectively) were used as receiver plants exposed to transgenic-plant-emitted volatiles [(*E*)-β-ocimene] under continuous air flow in open-flow chambers. In addition, greenhouse-based studies were similarly conducted in semi-natural conditions without climate control to assess the ability of inter-plant communication using transgenic plants for pest control. Such greenhouse trials with transgenic plants and the comparison of results with lab studies should be very useful for understanding the features of transgenic-plant-based pest control. Moreover, in addition to the above introduced communications between two plants, the potential of plant-plant-plant communications, in which there are chain-actions among three plants that play roles of 1) emitter, 2) receiver and then emitter, and 3) receiver, respectively, for the primed defense responses were evaluated.

## Materials and Methods

### Generation of transgenic tobacco plants

The full-length coding region of lima bean (*E*)-β-ocimene synthase (*PlOS*; EU194553) was inserted into the GFP reporter gene site of the binary vector pSMABR35SsGFP in which the selectable marker *bar* gene was replaced by a hygromycin phosphotransferase gene (*hpt*) [19]. The resulting plasmid, pSMAH-*PlOS*, was transformed into *Agrobacterium tumefaciens* strain EHA101 by electroporation. Tobacco (*Nicotiana tabacum* L. cv. SR1) plants that were aseptically grown from seeds for about 1 month were transformed via an *A. tumefaciens*-mediated leaf disc procedure [20], and selected using a medium containing 30 mg l^−1^ hygromycin. After rooting and acclimatization, the regenerated plants were grown in a closed greenhouse to set seeds. Sixteen lines of transgenic T_1_ seeds were tested for germination on 1/2 Murashige and Skoog medium supplemented with 30 mg l^−1^ hygromycin. T_2_ seeds harvested from each T_1_ individual plant that showed ca. 3∶1 segregation ratio were tested for hygromycin-resistance again. Finally, four homozygous T_2_ plant lines were used in further assays.

### Plants, herbivores and predators

Lima bean (*Phaseolus lunatus* cv. Pole Sieva) and maize (*Zea mays* L. cv. Royal Dent) plants were grown in a greenhouse. Each individual plant was grown in a plastic pot in a growth chamber at 25°C with a photoperiod of 16 h (natural+supplemental light), and used for the experiments by the time lima bean and maize were 2- and 1-week old, respectively. A wild-type (WT) or transgenic tobacco plant was grown in a plastic pot in a growth chamber at 25°C (160 μE m^−2^ s^−1^ during a 16-h photoperiod) for 4–6 weeks until it was ready to be used as an ‘emitter’.

Two-spotted spider mites (*Tetranychus urticae*) were obtained from the Laboratory of Ecological Information, Graduate School of Agriculture, Kyoto University, in 2002, and reared on kidney bean plants (*Phaseolus vulgaris* cv. Nagauzuramame) at 25°C with a 16 ∶ 8 h photoperiod. Predatory mites (*Phytoseiulus persimilis*) were purchased from Arysta Lifescience Corporation (Tokyo, Japan) in 2009. They were reared on *T. urticae* living on kidney bean plants under the same climate conditions as the spider mites.

*Mythimna separata* was transferred to our laboratory from a culture reared at the National Institute of Sericultural and Entomological Science in Tsukuba, Ibaraki, Japan, in 2001. The insects were reared on artificial diet (Insecta LF, Nihon Nousan Kogyo Ltd.) in the laboratory at 25°C with a 16 ∶ 8 h photoperiod. *Cotesia kariyai* was provided to the laboratory by Dr. Yooichi Kainoh at University of Tsukuba, Ibaraki, Japan. To maintain the wasp culture, 3rd to 4th instars of *M. separata* larvae were offered to female wasps for oviposition. Soon after emergence from their host, the wasp larvae span a cocoon. Clusters of cocoons were placed in a glass tube (*φ* = 22 mm, length = 200 mm) with honey as food for wasps at 18°C in the continuous dark conditions. The wasps emerging from the cocoons were kept (or stored) under the same conditions until they were used in the experiments within 7 days after the emergence. Oviposition-inexperienced females were used.

### Plant-plant communication assays

Air-flow experiments were conducted in polypropylene open-flow tunnels (40 cm wide, 80 cm long, 40 cm high) (Figure S1). Each tunnel was open at both ends and a fan was located at one of these openings to cause a continuous air flow (30 cm s^−1^) that flowed across from ‘emitter’ to ‘receiver’ plants through the tunnel. Four tobacco plants (WT or NtOS2 plants [emitter plants]) and four lima bean or maize (receiver plants) were placed 30 cm apart for 3 days. Hereafter, the receiver plants are referred to as VOCos-receivers and VOCwt-receivers, according to the emitter plants' characteristics (NtOS7 or WT). During treatments, the temperature was maintained at 25°C with a photoperiod of 16 h. The light period was from 7 AM to 11 PM. After 3 days, the plants were used for herbivore treatments.

Greenhouse experiments using the system called Development and Assessment of Sustainable Humanosphere System located at the Uji Campus of Kyoto University were performed during March 2010–June 2010 and September 2010–March 2011. Four tobacco emitter plants (WT or NtOS2 plants) and four receiver lima bean plants were placed 30 or 60 cm apart in a chamber (200 cm wide, 300 cm long, 450 cm height) of the greenhouse, without climate control or air flow. During the 7-day treatments, the temperature ranged from 24 to 36°C during the day and from 16 to 24°C at night with an average day period from 5:30 AM to 6:30 PM. Each fumigation was independently replicated 4 times for a given set of experiments.

### Plant-plant-plant communication assays

The complete assay was conducted as a two-part plant-plant communication assay in a greenhouse (see above). Four VOCos-receiver or VOCwt-receiver lima bean plants (the first receiver plants) were placed 30 cm apart from four emitter tobacco plants (NtOS2 or WT plants) in the greenhouse for 7 days. The first receiver plant was subsequently treated with 40 *T. urticae* and used as ‘emitter’ for the second assay started sequentially. During the second assay, four uninfested lima beans (the second receiver plants) were placed 30 cm apart from the four VOCos-receiver or VOCwt-receiver plants in a greenhouse for 1 or 7 days. The second receiver was subsequently exposed to 40 *T. urticae* for 1 day and subjected to use. Throughout the second assays, transgenic and WT tobacco plants were removed to avoid the effect of their volatiles. Each fumigation was independently replicated 4 times for a given set of experiments.

### Reverse transcription (RT)-PCR and real-time PCR

Total RNA was isolated from leaf tissues using a Qiagen RNeasy Plant RNA kit and an RNase-Free DNase Set (Qiagen) following the manufacturer's protocol. First-strand cDNA was synthesized using Takara PrimeScript RT reagent Kit with 0.5 µg of total RNA (see above) at 37°C for 15 min and 85°C for 5 sec. Real-time PCR was done on an Applied Biosystems 7300 Real Time PCR System using Power SYBR® PCR Master Mix (Applied Biosystems), cDNA (1 µl from 10 µl of each RT product pool), and 300 nM primers. The following protocol was followed: initial polymerase activation: 2 min at 50°C and 10 min at 95°C; 40 cycles of 15 s at 95°C and 60 s at 60°C. PCR conditions were chosen by comparing threshold values in a dilution series of the RT product, followed by non-RT template control and non-template control for each primer pair. Relative RNA levels were calibrated and normalized with the level of an actin gene (GQ281246) mRNA. Primers used were as follows: *PlOS* (5′-CAACAATGCATGGGTCTCAG-3′ and 5′-TGCTGCTTCCCCTCTCTCTA-3′) and an actin gene (5′-CTGGAATGGTTAAGGCTGGA-3′ and 5′-CAATTGCTAACGATTCCGTGT-3′).

### Volatile analysis

The receiver lima bean plant or maize plant in a pot was infested with 40 adult female *T. urticae* and 4 third instar *M. separata* larvae for 24 h. An uninfested potted plant immediately or 1 day after exposure to VOCs served as control. We collected the volatiles from a potted plant in a glass container using 100 mg of Tenax-TA resin (20/35 mesh; GL Science, Japan) packed in a glass tube (3.0 mm i.d., 160 mm length) for 2 h, at a flow rate of 100 ml min^−1^ with clean air. *n*-Tridecane (0.1 µg) was also added to the glass container as an internal standard. The volatile compounds collected were analyzed by gas chromatography-mass spectrometry (GC-MS) according to the method described in [21]. The headspace volatiles were identified by comparing their mass spectra and retention times to those of authentic compounds, and quantified with calibration curves made using authentic compounds. (*E*)-β-Ocimene was quantified using its authentic compound (SAFC).

### Olfactometer assay for predatory mite *P. persimilis*

A Y-tube olfactometer was used to test the olfactory responses of adult female predatory mites (*P. persimilis*) to plant volatiles [22]. The mites were individually introduced at the start point on a steel wire in a laboratory room at 25°C. The behavior of the mite on the wire was observed until the mite reached the far end (finish line) of one of the arms. The connections of the odor-source containers to the olfactometer arms were exchanged every five bioassays. Assays using 20 or 10 predators were carried out as a single replicate in a day. Three or four replications (i.e. 70–80 predators in all) were carried out on different days. The results from 3 or 4 replications of each experiment were subjected to a binomial test to evaluate whether the result in each experiment differed from the null hypothesis in which predators showed a 50∶50 distribution between the two odor sources. Predators that did not pass the finish line of either arm within 5 min (“no choice” subjects) were excluded from the statistical analysis.

### Flight responses of parasitoids to maize plants

The flight responses of female *C. kariyai* were observed with respect to two groups of maize plants. Each respective group consisting of three potted plants was positioned 20 cm apart in a cage (25×35×30 cm; three windows covered by nylon gauze and one door for introducing plants and wasps). An individual female wasp was released halfway between the two groups. Pots in the cage were replaced every 10 trials. The first landing by each wasp on a plant in either of the two pots was recorded as its choice. The wasp—once it landed on a plant—was immediately removed from the cage with an insect aspirator. Three replicates were performed, each with 10 wasps per test. Wasps that did not land on either of the two pots within 20 min (“no choice” subjects) were excluded from the statistical analysis (binomial test). Each bioassay was performed in a climate-controlled room at 25°C.

### Performance of *T. urticae* females

A *T. urticae* adult female was transferred onto a VOCos/VOCwt-exposed lima bean leaf square (10×10 mm) on a wet cotton-laid petri dish (90 mm diameter). Each dish containing 10 leaf squares was incubated in a climate-controlled room at 25°C with a photoperiod of 16 h, and the number of eggs laid by each female was counted every 24 hours for up to 3 days.

### Performance of *M. separata* larvae

Third instar *M. separata* larvae (each 3+/−1 mg) were released onto a VOCos/VOCwt-exposed maize plant in a pot. Each plant with larvae was incubated in a climate-controlled room at 25°C with a photoperiod of 16 h, and the larvae were collected and weighed every 24 hours for up to 3 days.

## Results

### Generation of transgenic tobacco plants emitting (*E*)-n-ocimene

**Figure 1:**
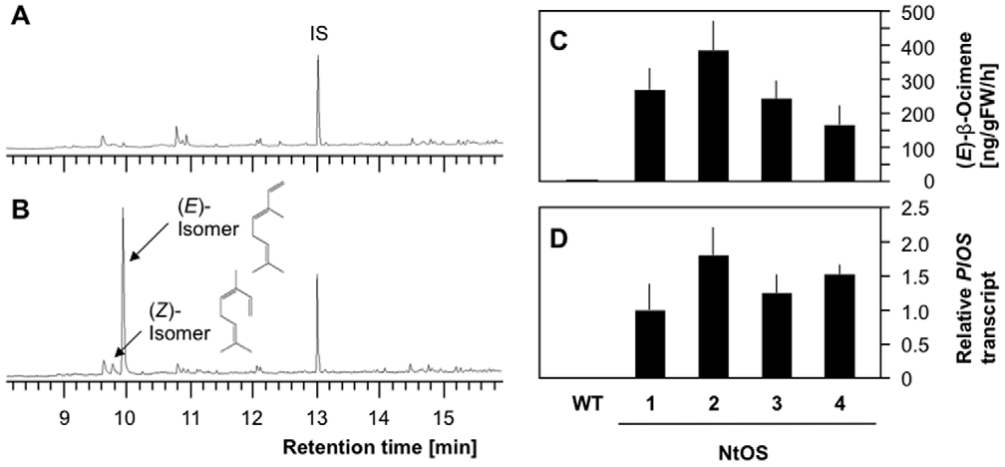
Emission of β-ocimene from *PlOS*-transgenic plants. Representative gas chromatography-mass spectrometry profile of volatiles emitted from the wild-type (**A**) and transgenic (**B**) tobacco plants are presented. Values for (*E*)-β-ocimene emission (**C**) and relative *PlOS* transcript (**D**) in transgenic plants represent the means + standard errors (n = 4–5).

In order to assess the impact of manipulated VOCs on plant-plant communications, we generated transgenic plants that constitutively biosynthesize and emit VOCs. We prepared gene constructs consisting of lima bean (*E*)-β-ocimene synthase *PlOS* [23] inserted downstream of the constitutive 35S cauliflower mosaic viral (CMV) promoter, and successfully generated a set of independent transgenic tobacco lines. Four representative examples exhibiting substantial trans-gene (*PlOS*) expression and (*E*)-β-ocimene emission are shown in Figure 1. Their emissions ranged between 166 ng [gram fresh weight (gFW h^−1^) (NtOS4) and 384 ng [gFW h^−1^] (NtOS2), whereas WT tobacco plants emitted these compounds at only extremely low levels (∼2 ng [gFW h^−1^]) (Figure 1C). The levels of (*E*)-β-ocimene emitted by the above transgenic plants were sufficiently relevant to those emitted by WT lima bean plants: e.g., about 3,000 ng [gFW h^−1^] and 30 ng [gFW h^−1^] in response to feeding by *Spodoptera littoralis* [23] and *T. urticae* (see below), respectively. In addition, none of the transgenic lines exhibited any differences in their detectable morphology or their levels of emission of VOCs other than (*E*)-β-ocimene.

Only a trace amount of the (*Z*)-isomer of β-ocimene (5% of the total β-ocimene products, Figure 1B) was included in all the headspace volatiles, and this percentage corresponded to the composition of β-ocimene isomers generated from the recombinant *PlOS* protein expressed in *Escherichia coli* using geranyl diphosphate as a substrate [23]. A similar result was also observed in a preliminary study in transgenic torenia plants expressing *PlOS* (Shimoda *et al.* unpublished).

### Direct defenses of bean and maize plants induced by transgenic-plant-emitted volatiles (VOCos)

**Figure 2:**
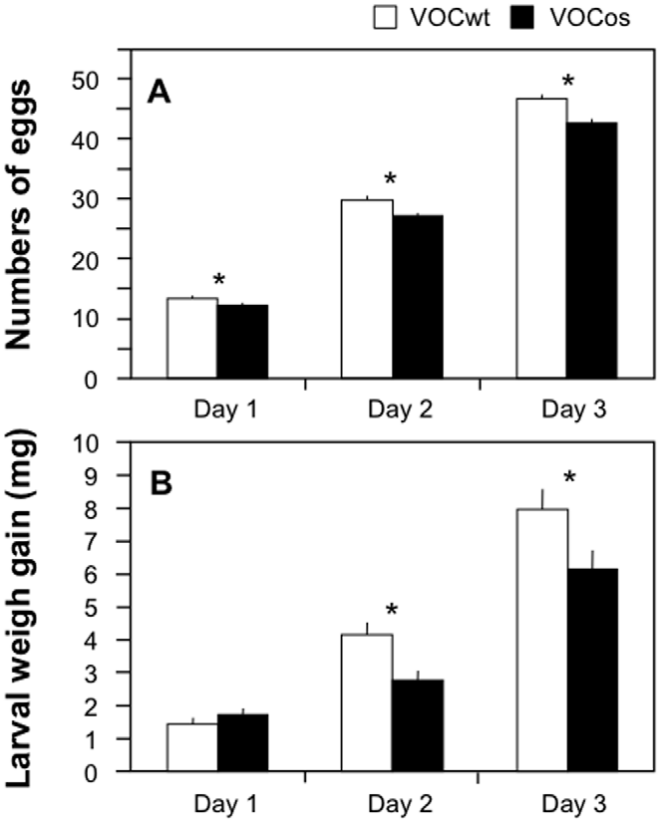
VOC-primed defense responses of receiver plants to damage by *T. urticae* females (A) or *M. separata* larvae (B). Plants were exposed to VOCs emitted from either WT (VOCwt) or NtOS2 (VOCos) tobacco plants for 3 days in an open-flow tunnel. The number of eggs laid by *T. urticae* on the leaf section of lima beans or *M. separata* larval weight gained on maize plants was determined every 3 days after the end of exposure. Data represent the mean + standard errors (n = 43–49 for *T. urticae* and n = 23–24 for *M. separata*). Asterisks indicate significant differences between VOCwt and VOCos (Student's t-test, P<0.05).

Under a continuous air flow, lima bean or maize receiver plants (hereafter referred to as VOCos-receivers) were placed downwind of NtOS2 plants for 3 days (Figure 2). The crops placed downwind of WT tobacco plants served as controls (VOCwt-receivers). Since inter-plant communications are known to occur only at short distances [24], [25], we separated ‘receiver’ and ‘emitter’ plants by a short distance (∼30 cm, see Figure S1). After exposure, a *T. urticae* adult female was placed on the leaf sections of the receiver lima bean plants, and the oviposition of her eggs was counted every 24 hours for up to 3 days (Figure 2A). VOCos-receiver bean leaves exhibited a lower rate of oviposition of *T. urticae* than the VOCwt-receiver leaves for up to 3 days (*P*<0.05, Student's t-test). Likewise, lower weight gain of common armyworm (*M. separata*) larvae was observed on maize plants 2 and 3 days after exposure to VOCos, compared to those exposed to VOCwt (*P*<0.05, Student's t-test; Figure 2B).

### Priming of indirect defenses of plants by VOCos

**Figure 3:**
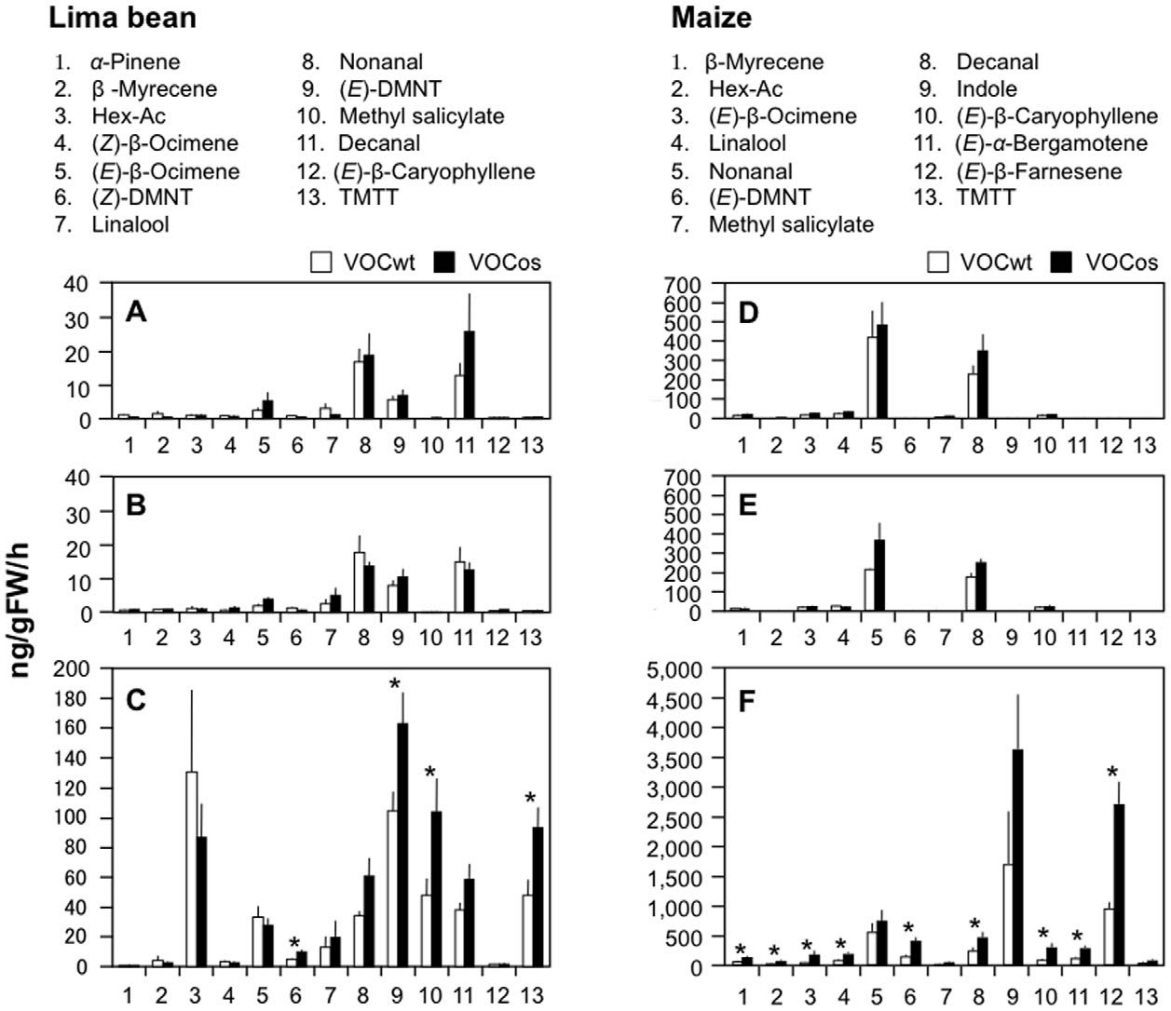
VOC emissions from receiver lima bean or maize plants in an open-flow tunnel. VOCs were collected from receiver lima bean or maize plants immediately after exposure to either VOCwt or VOCos (**A**, **D**), and after receiver plants were subjected to 1 day post-herbivory by *T. urticae* (**C**) or *M. separata* (**F**). Uninfested plants incubated for 1 day after VOC exposure served as controls (**B**, **E**). Data represent the mean + standard errors (n>4). Asterisks indicate significant differences from control volatiles (Student's t-test, P<0.05).

Next we measured the emission level of VOCs in VOCos-receiver lima bean and maize plants. It was found that neither of these VOCos-receiver plants was stimulated to release VOCs, compared with VOCwt-receivers (Figures 3A and D). However, VOCos-receiver bean plants following infestation of *T. uriticae* for 1 day emitted higher levels of VOCs, including methyl salicylate (MeSA) and homoterpenes ((*E*)-4,8-dimethyl-1,3,7-nonatriene [(*E*)-DMNT] and its isomer (*Z*)-DMNT, and (*E*,*E*)-4,8,12-trimethyltrideca-1,3,7,11-tetraene [TMTT]), compared to the *T. urticae*-infested VOCwt-receiver plants (*P*<0.05, Student's t-test, see Figure 3C). Similar results were obtained when maize plants were infested with *M. separata* after VOCos exposure: the plants emitted higher levels of VOCs, including β-myrcene, Hex-Ac, (*E*)-β-ocimene, linalool, (*E*)-DMNT, decanal, (*E*)-β-caryophyllene, (*E*)-α-bergamotene and (*E*)-β-farnesene, compared to the VOCwt-receiver plants infested with *M. separata* (Figure 3F). The emission levels were, however, not enhanced when VOCos-receiver bean or maize plants were kept without infestation for 1 day after the exposure (Figures 3B and E).

The emission levels of (*E*)-β-ocimene were very slightly increased in VOCos-receiver bean plants analyzed immediately after VOCos exposure (*P* = 0.27, Student's t-test; Figure 3A), which was probably due to re-emission of the volatiles adsorbed onto waxy layers on the plants' surface during the exposure. However, this was not observed in VOCos-receiver maize plants.

**Figure 4:**
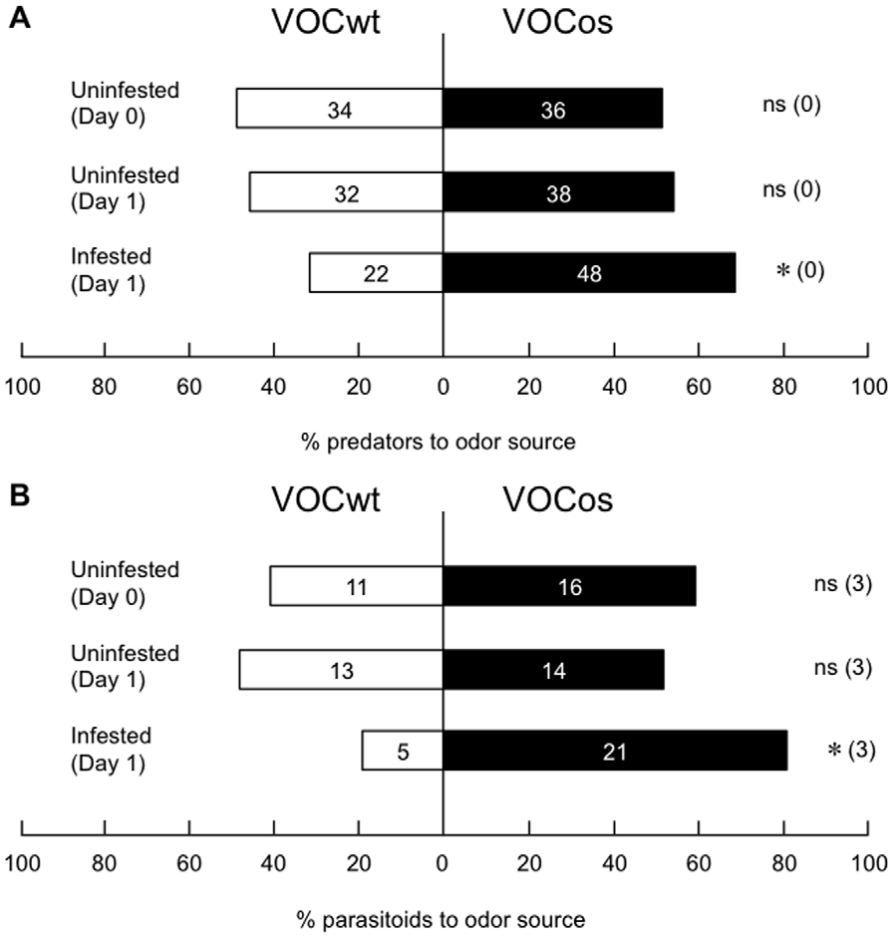
Indirect defenses of VOCos/VOCwt-exposed plants. (**A**) Olfactory response of carnivorous predatory mites (*P. persimilis*) when offered VOCwt-exposed vs. VOCwt-exposed lima bean receiver plants. (**B**) The flight responses of parasitoids (*C. kariyai*) when offered VOCwt-exposed vs. VOCos-exposed receiver maize plants. The receiver plants were assessed immediately (day 0) after exposure to either VOCwt or VOCos, and subjected to one day post-herbivory by *T. urticae* (**A**) or *M. separata* (**B**). Uninfested plants incubated for 1 day after VOC exposure served as controls. The bar represents the overall percentages of arthropods choosing either of the odor sources. The figures in parentheses represent the numbers of predators that did not choose either odor source (no choice subjects). A binomial test was conducted to evaluate whether the result in each experiment differed from the null hypothesis in which predators showed a 50∶50 distribution between the two odor sources (*: *P*<0.05, ns: *P*>0.05).

It has been reported that the entire blend of lima bean volatiles including TMTT and MeSA plays an important role in the attraction of predatory mites (*Phytoseiulus persimilis*) [26], [27]. Moreover, Arabidopsis plants emitting (3*S*)-(*E*)-nerolidol and (*E*)-DMNT] are able to attract carnivorous predatory mites [28]. Based on these facts we compared the olfactory response of predatory mite *P. persimilis* between lima bean plants exposed to VOCos and VOCwt followed or not by *T. urticae* infestation (Figure 4A). As expected, VOCos-receiver plants fed upon by *T. urticae* were preferred by *P. persimilis*, compared to the infested VOCwt-receiver plants (*P*<0.05, binomial test). In contrast, the predatory mites did not unambiguously discriminate volatiles of VOCos-receiver bean plants from those of the VOCwt-receiver plants in the uninfested condition (Figure 4A). Similar effects were observed on the flight responses of *C. kariyai* females to the two groups of VOCos-receiver and VOCwt-receiver maize plants in a cage. The parasitoids did not discriminate volatiles of VOCos-receiver plants from those of VOCwt-receiver plants in the uninfested conditions but started to prefer the VOCos-receiver plants relative to VOCwt-receiver plants after *M. separata* infestation (Figure 4B).

### VOCos-primed defense responses in greenhouse conditions

We next conducted experiments in a greenhouse. Hereafter, we focus on the lima bean system as a model case. As in the above open-flow system, uninfested lima bean plants were placed 30 cm apart from NtOS2 or WT plants (VOCos-receivers or VOCwt-receivers) in a greenhouse for 7 days without any climate control. We then evaluated oviposition of *T. urticae* eggs on the VOCos-receiver or VOCwt-receiver leaf sections and their capability of indirect defense by analyzing the emission levels of VOCs and the attraction of *P. persimilis* after *T. urticae* attack for 1 day.

**Figure 5:**
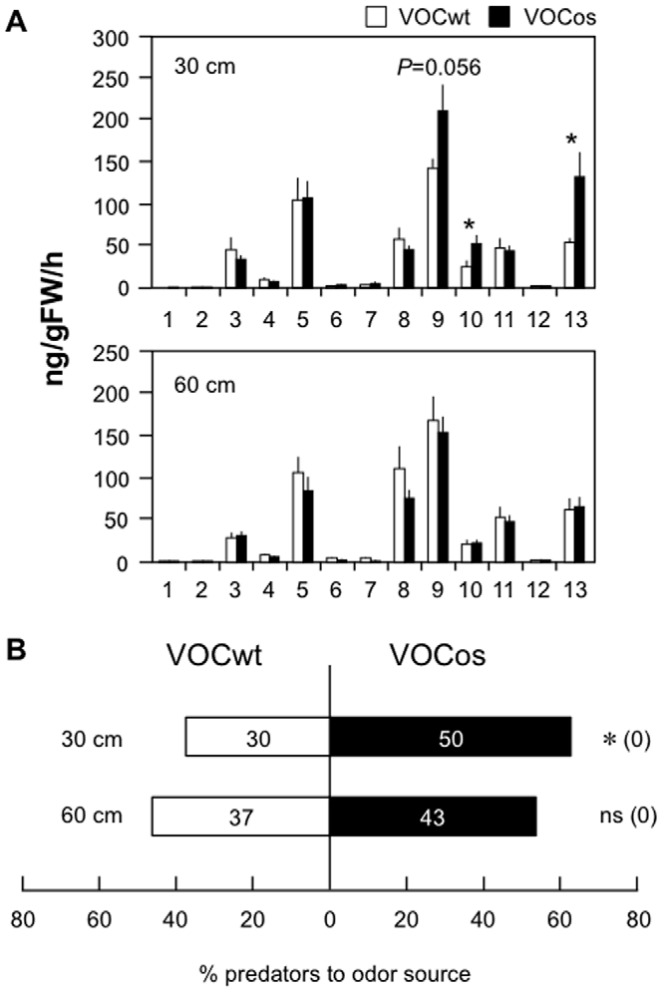
Indirect defenses of VOCos/VOCwt-exposed lima bean plants in greenhouse conditions. Uninfested plants were placed 30 or 60 cm apart from NtOS2 (VOCos) or WT (VOCwt) in a greenhouse for 7 days. The receiver plants were treated with *T. urticae* for 1 day and subjected to VOC collections (**A**) and olfactory assays of predatory mites (**B**). Asterisks indicate significant differences from control volatiles (Student's t-test, *P*<0.05). For more information, see Figures 3 and 4.

In contrast to the results using an open-flow system, the oviposition of *T. urticae* eggs was not different between the VOCos-receiver and VOCwt-receiver leaves in a greenhouse (Figure S2). On the other hand, the VOCos-receiver plants emitted higher levels of TMTT and MeSA in response to *T. urticae* attack, causing more attraction of predatory mites, in comparison to the VOCwt-receiver plants, in the greenhouse (Figure 5). When VOCos-receiver plants were placed 60 cm apart, the priming of indirect defenses was not detected.

### Plant-plant-plant communications

As shown in Figure 5A, VOCos-receiver bean plants in a greenhouse showed higher levels of VOCs after *T. urticae* attack, in comparison to the infested VOCwt-receiver plants. These findings suggested that such high levels of the emitted VOCs may also affect the neighboring plants (the second receiver plants), leading to more efficient indirect plant defenses. Accordingly, we conducted experiments to assess the priming effect on indirect defenses in uninfested lima beans placed near either the VOCos-receiver or VOCwt-receiver plants.

**Figure 6:**
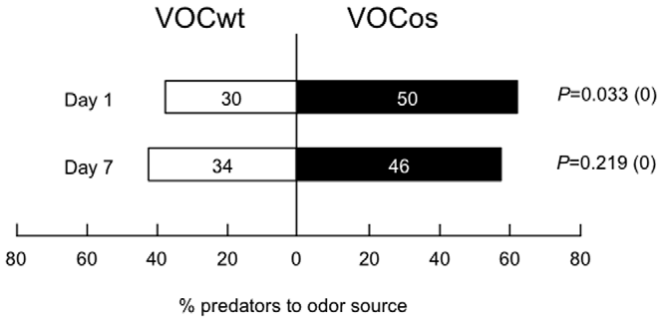
Plant-plant-plant communications for indirect defenses. VOCos-receiver or VOCwt-receiver lima bean plants were placed 30 cm apart from four emitter tobacco plants (NtOS2 or WT plants) in a greenhouse for 7 days. The VOCos-receiver or VOCwt-receiver plant was subsequently treated with *T. urticae*, and uninfested plants were placed 30 cm apart from the VOCos-receiver or VOCwt-receiver plants for 1 or 7 days. The second receiver was subsequently exposed to *T. urticae* for 1 day.

In response to *T. urticae* infestation, the second receiver plants that had been placed 30 cm apart from the infested VOCos-receiver plants for 1 day were more attractive to *P. persimilis*, in comparison to those that had been placed near the infested VOCwt-receiver plants (*P*<0.05, binomial test; Figure 6). However, when the second receiver plants had been kept with the infested VOCos-receiver plants for 1 week, *P. persimilis* was less attracted (*P* = 0.22, binomial test; Figure 6).

## Discussion

In the current study, we found that transgenic-plant-volatiles (VOCos), which included (*E*)-β-ocimene, from *PlOS*-overexpressing plants, primed defense responses to herbivory in eavesdropping bean and maize plants. We examined these responses under two experimental conditions using an “open-flow chamber” and a “greenhouse without climate control”. These experiments confirmed the importance of relying on realistic experimental conditions for plant-plant communication studies [29]. Firstly, in our “open-flow” condition, both induced direct and indirect defenses (reflected by reduced development/reproduction and attraction of predators) were found to be primed by exposure to VOCos. These results were expected because a variety of plant species, including lima bean and maize, exhibit potent responses to VOCs [3], [7], [9]–[11]. Moreover, several angiosperm plants have been reported to show enhanced direct and indirect defenses upon VOC-induced priming, following the transcriptional regulation of genes that mediate oxylipin signaling and defense responses [10], [11]. Continuous air-pump and open-flow chamber systems would be useful for clarifying the mechanisms of plant-plant interactions via genetic manipulation and biochemical and ecological analyses.

In addition, studies examining the mechanisms and ecological consequences of such interactions should be conducted by working in different conditions (lab and field) [2]. At this point, there is general agreement that volatile communication occurs in the field for at least some plant systems and that several different signaling pathways are involved in laboratory experiments. Accordingly, we shifted from the “open-flow chamber” to a greenhouse study during the current study. To the best of our knowledge, there has been no similar attempt at combining the two systems. Although it would be best to conduct studies in the field, studies cannot be conducted with *genetically modified* (GM) plants in the field in Japan. This restriction, in turn, encouraged us to carry out greenhouse experiments, but without climate control. In the greenhouse experiments, it was found that *T. urticae* females laid their eggs on VOCos-receiver lima bean leaves at the same level as on VOCwt-receiver leaves (Figure S2). This was probably due to the weaker atmospheric impact of airborne signals in the greenhouse (without air-flow) compared to the open-flow system (with air-flow). These findings are in line with the report from [30] that β-ocimene has no detectable effect on the priming or induction of the secretion of extrafloral nectar in naturally growing lima bean tendrils. Therefore, different environments (open-flow chamber<greenhouse<field) seem to differently affect whether and how receiver plants respond by undertaking defensive actions.

Indirect defenses carried out by the attracted predatory mites were strengthened in VOCos-receiver plants in response to *T. urticae* infestation. The indirect defense against *T. urticae* was exhibited only within a distance of 30 cm from NtOS2 (Figure 5), as predicted from reports showing that inter-/intra-plant communications occur at proximate distances [24], [25].

As shown in Figure 6, communication among three plants via VOCs was found here for the first time. This plant communication occurred especially when the second receiver plants were exposed to *T. urticae*-induced volatiles emitted from VOCos-receiver plants for 1 day. These results clearly suggest that VOCos-receiver plants have two potential means for the priming of defense responses, i.e., by interacting with herbivores' enemies and with neighboring conspecific plants by releasing a VOC blend. Although we do not know the ecological consequences of such multi-plant communication, plants may have evolved this system that allows plants to emit and respond to volatile cues in order to sustain their population built up of genetically identical plants. This view is supported by recent findings indicating that communication is more effective between branches that are genetically identical than between branches that are genetically different [31]. Nevertheless, it was reported that resistance-inducing volatiles of lima bean move over distances at which most leaves that can receive the signal still belong to the same plant [25]. Therefore, VOC signals that are responsible for communication between individuals are also required for coordination among branches within a single plant [7], [24], [32]. Future studies should provide considerable insight into the factors that enable a volatile communication system in individual plants and their population.

## Supporting Information

Figure S1 **Schematic drawing of experimental set-up for inter-plant communication assay.**
(TIF)

Figure S2 **VOC-primed defense responses in receiver lima bean plants to damage by** ***T. urticae*** **in greenhouse conditions.** Uninfested plants were placed 30 cm (**A**) or 60 cm (**B**) apart from NtOS2 (VOCos) or WT (VOCwt) in a greenhouse for 7 days. The number of eggs laid by a *T. urticae* female on a receiver leaf section was determined for up to 3 days. Data represent the mean + standard errors (n = 41–47). Asterisks indicate significant differences between VOCwt and VOCos (Student's t-test, *P*<0.05).
(TIF)
