# Supplementary figures and images for: The Composite Effect of Transgenic Plant Volatiles for Acquired Immunity to Herbivory Caused by Inter-Plant Communications
Source: PLoS One. 2011 Oct 12;6(10):e24594. doi: 10.1371/journal.pone.0024594 (PMC3192036; doi:10.1371/journal.pone.0024594)

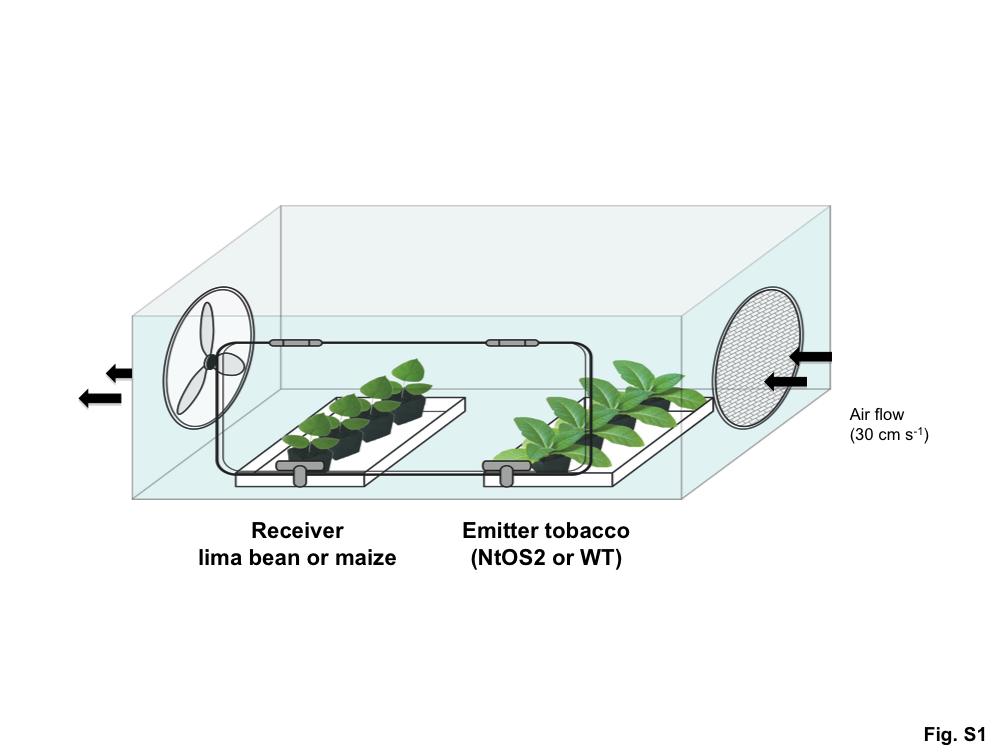

Supplement: Figure S1 — Schematic drawing of experimental set-up for inter-plant communication assay. (TIF) [file pone.0024594.s001.tif]

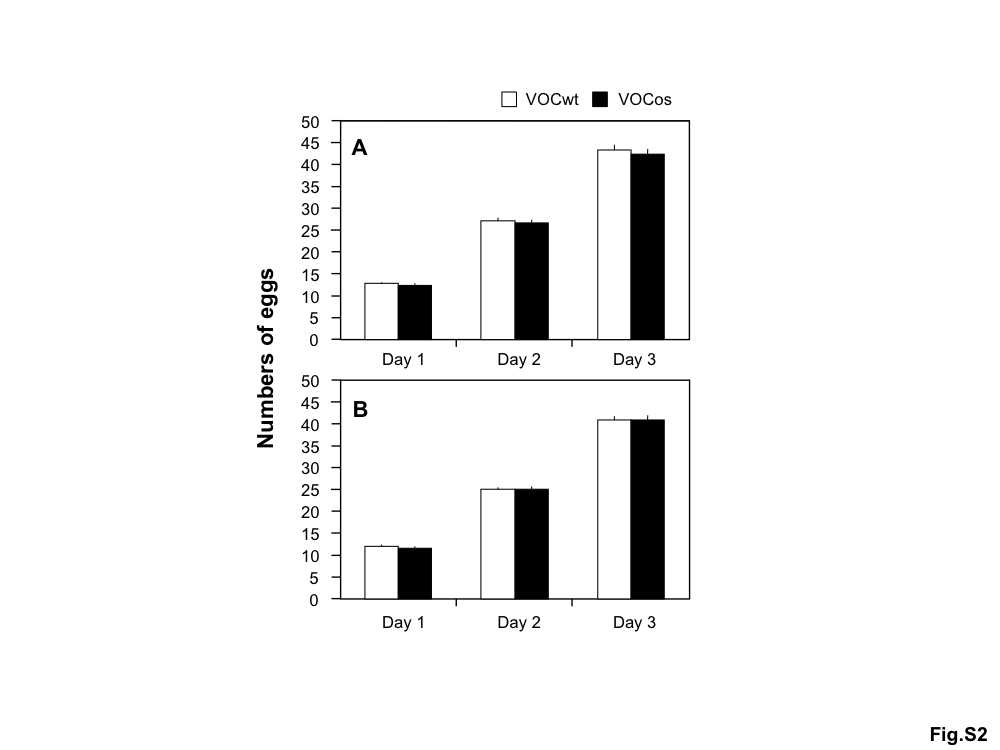

Supplement: Figure S2 — VOC-primed defense responses in receiver lima bean plants to damage by T. urticae in greenhouse conditions. Uninfested plants were placed 30 cm (A) or 60 cm (B) apart from NtOS2 (VOCos) or WT (VOCwt) in a greenhouse for 7 days. The number of eggs laid by a T. urticae female on a receiver leaf section was determined for up to 3 days. Data represent the mean + standard errors (n = 41–47). Asterisks indicate significant differences between VOCwt and VOCos (Student's t-test, P<0.05). (TIF) [file pone.0024594.s002.tif]
